# Supplementary material for: Meta-Analysis and Gene Set Enrichment Relative to ER Status Reveal Elevated Activity of MYC and E2F in the “Basal” Breast Cancer Subgroup
Source: PLoS One. 2009 Mar 9;4(3):e4710. doi: 10.1371/journal.pone.0004710 (PMC2650420; doi:10.1371/journal.pone.0004710)
Supplement: Dataset S1 — GSEA results from first screen. The file contains a zipped archive of the HTML results of the second GSEA screen. Reports for enriched or depleted datasets may be accessed from the “index.html” link within the my_analysis.GseaPreranked.1219975950625.rpt.zip ZIP archive. Enrichment plots are available for each of the 50 most enriched and depleted gene sets. (13.32 MB ZIP) [file pone.0004710.s001.zip › my_analysis.GseaPreranked.1219975950625.rpt.zip!//TSA_HEPATOMA_CANCER_UP.html]

Details for gene set TSA\_HEPATOMA\_CANCER\_UP[GSEA]

|  || Dataset | zSco.ERneg.to.ERpos\_collapsed |
| Phenotype | NoPhenotypeAvailable |
| Upregulated in class | na\_neg |
| GeneSet | TSA\_HEPATOMA\_CANCER\_UP |
| Enrichment Score (ES) | -0.42199868 |
| Normalized Enrichment Score (NES) | -1.6120584 |
| Nominal p-value | 0.012903226 |
| FDR q-value | 0.11851024 |
| FWER p-Value | 0.998 |
Table: GSEA Results Summary

  

Fig 1: Enrichment plot: TSA\_HEPATOMA\_CANCER\_UP      
 Profile of the Running ES Score & Positions of GeneSet Members on the Rank Ordered List

  

| PROBE | GENE SYMBOL | GENE\_TITLE | RANK IN GENE LIST | RANK METRIC SCORE | RUNNING ES | CORE ENRICHMENT || 1 | CAV2 |  |  | 730 | 3.881 | -0.0019 | No |
| 2 | FBN2 |  |  | 862 | 3.683 | 0.0386 | No |
| 3 | KRT7 |  |  | 1262 | 3.180 | 0.0521 | No |
| 4 | RHOG |  |  | 1910 | 2.600 | 0.0389 | No |
| 5 | TP53I11 |  |  | 2192 | 2.391 | 0.0505 | No |
| 6 | CD8B |  |  | 2479 | 2.211 | 0.0592 | No |
| 7 | CDKN2C |  |  | 3148 | 1.866 | 0.0344 | No |
| 8 | HBEGF |  |  | 4692 | 1.255 | -0.0646 | No |
| 9 | ANK1 |  |  | 4791 | 1.226 | -0.0552 | No |
| 10 | TGFB1 |  |  | 5452 | 0.993 | -0.0913 | No |
| 11 | EFNB3 |  |  | 5674 | 0.923 | -0.0953 | No |
| 12 | FASTK |  |  | 5913 | 0.844 | -0.1017 | No |
| 13 | TRIP6 |  |  | 6573 | 0.627 | -0.1427 | No |
| 14 | ITGA7 |  |  | 7035 | 0.475 | -0.1710 | No |
| 15 | MMP13 |  |  | 7376 | 0.355 | -0.1917 | No |
| 16 | MMP19 |  |  | 7676 | 0.239 | -0.2109 | No |
| 17 | TIMP2 |  |  | 8021 | 0.096 | -0.2355 | No |
| 18 | NGFR |  |  | 8219 | 0.029 | -0.2500 | No |
| 19 | GLRX |  |  | 8308 | -0.001 | -0.2566 | No |
| 20 | VTN |  |  | 9295 | -0.454 | -0.3246 | No |
| 21 | CTGF |  |  | 9358 | -0.487 | -0.3226 | No |
| 22 | CD36 |  |  | 9597 | -0.616 | -0.3321 | No |
| 23 | CASP9 |  |  | 10260 | -0.998 | -0.3683 | No |
| 24 | VEGFC |  |  | 10580 | -1.225 | -0.3755 | No |
| 25 | BSG |  |  | 11198 | -1.743 | -0.3982 | Yes |
| 26 | PLA2G6 |  |  | 11400 | -1.936 | -0.3868 | Yes |
| 27 | JUP |  |  | 11461 | -1.998 | -0.3640 | Yes |
| 28 | JUNB |  |  | 11504 | -2.039 | -0.3393 | Yes |
| 29 | QSCN6 |  |  | 11754 | -2.269 | -0.3270 | Yes |
| 30 | CD9 |  |  | 11784 | -2.309 | -0.2976 | Yes |
| 31 | COL1A2 |  |  | 12131 | -2.748 | -0.2860 | Yes |
| 32 | ITGA3 |  |  | 12256 | -2.918 | -0.2555 | Yes |
| 33 | MMP11 |  |  | 12368 | -3.051 | -0.2221 | Yes |
| 34 | FOSB |  |  | 12385 | -3.084 | -0.1811 | Yes |
| 35 | ARHGDIB |  |  | 12440 | -3.165 | -0.1419 | Yes |
| 36 | LUM |  |  | 12475 | -3.221 | -0.1004 | Yes |
| 37 | BAD |  |  | 12744 | -3.724 | -0.0696 | Yes |
| 38 | PDGFRB |  |  | 12886 | -4.104 | -0.0241 | Yes |
| 39 | IGFBP2 |  |  | 12897 | -4.147 | 0.0318 | Yes |
Table: GSEA details [plain text format]

  

Fig 2: TSA\_HEPATOMA\_CANCER\_UP: Random ES distribution      
 Gene set null distribution of ES for **TSA\_HEPATOMA\_CANCER\_UP**
